# Microbial diversity and metabolic potential in long-term Cr(VI) polluted soil during in situ biostimulation: a pilot effective assay

**DOI:** 10.1007/s11356-025-36804-7

**Published:** 2025-08-11

**Authors:** Fanny A. Flores-Gallegos, Fernando García-Guevara, Leticia Vega-Alvarado, Paloma Lara, Verónica Jiménez-Jacinto, Katy Juárez

**Affiliations:** 1 Instituto de Biotecnología, Universidad Nacional Autónoma de México, Cuernavaca, Morelos México; 2 Centro de Ciencias Genómicas, Universidad Nacional Autónoma de México, Cuernavaca, Morelos México; 3 Instituto de Ciencias Aplicadas y Tecnología, Universidad Nacional Autónoma de México, Ciudad Universitaria, Ciudad de Mexico, México

**Keywords:** Chromium, Biostimulation, Soil bacterial diversity, Molasses, Metatranscriptome, Bioremediation, Cr(VI), Halomonas

## Abstract

Excess industrial Cr(VI) waste and its improper disposal have resulted in the contamination of diverse environments, including soils and aquifers. To contend with high concentration of Cr(VI), a dangerous mutagen and oxidizing agent, diverse bacteria have developed a broad spectrum of metabolic strategies, mainly through chromate efflux pumps and reduction of Cr(VI) to Cr(III), which is less toxic and unable to cross biological membranes. In this study, we performed an in situ biostimulation assay in a highly alkaline and saline soil from a long-term contaminated site in Guanajuato, México. Four percent molasses was an effective treatment in promoting the Cr(VI) reduction by indigenous microorganisms. Initial Cr(VI) concentration was 5.6 to 12.4 g per kg of soil; After biostimulation assay (20 days), Cr(VI) was reduced from 0.75 to 3.02 g per kg of soil. DNA and RNA extraction from biostimulated samples was performed to carry out metagenomic and metatranscriptomic studies. Furthermore, 16S rDNA V3 and V4 amplicons were sequenced using illumina MiSeq technology complementing the study. The results showed an enrichment at Class level of *Gammaproteobacteria, Alphaproteobacteria, Actinobacteria, Nitriliruptoria* and *Bacilli.* The enrichment of *Halomonas spp*. during the biostimulation assay was remarkable, reaching 92% of the population and becoming the most dominant genus. On the other hand, comparative metagenomic and metatranscriptomic analysis was carried out in order to know the whole microbial population and the genes expressed during the reduction of Cr(VI) to Cr(III). We identified reductase genes associated with various bacterial groups. Interestingly, all the expressed reductase genes were exclusively from the genus *Halomonas*, which are related with our taxonomic assignment analysis. This study improves our understanding of the response of bacterial communities to high exposure to chromate and offers an alternative to the restoration of environments severely contaminated with this powerful toxic agent.

**Supplementary Information:**

The online version contains supplementary material available at 10.1007/s11356-025-36804-7.

## Introduction

Microbial diversity in soil is one of the critical factors for maintaining a healthy and dynamic ecosystem. Native microbiota play a key role in various processes, including biogeochemical cycles, soil fertility, and detoxification (Yang et al. 2024; Delgado-Baquerizo et al. 2016; Madsen 2011; Barrios 2007). However, each site presents unique characteristics and challenges (such as pH, nutrient availability, and the presence of heavy metals) that influence bacterial community composition (Liu et al. 2023; Hermans et al. 2017).

The impact of anthropogenic activities on the structure of indigenous bacterial communities has been extensively studied (Lauber et al. 2008; Li et al. 2021; Wu et al. 2022). It has been observed that the physicochemical characteristics of the soil strongly influence the composition of these communities, affecting the distribution and abundance of different bacterial species (Lauber et al. 2008). Improper disposal of industrial waste has increased the accumulation of toxic compounds in the environment, which can reduce microbial diversity. However, this diversity could potentially be restored through appropriate decontamination processes (Wu et al. 2022).

Heavy metals are major contributors to environmental pollution, and chronic exposure to elements such as chromium, manganese, nickel, lead, and cadmium poses significant health risks to humans (Fu and Xi 2019; Zhitkovich 2011). Chromium has been widely used in various industrial processes (including tannery, electroplating, and pigment manufacturing) for several decades. However, the mismanagement of waste and residues from these activities has led to severe pollution issues (Zhao et al. 2022).

Chromium oxidation states range from -II to VI, with hexavalent chromium (Cr(VI)) and trivalent chromium (Cr(III)) being the most stable species in the environment Kotasâ and Stasicka 2000; Losi et al. 1994). Cr(VI) is highly toxic, carcinogenic, and teratogenic, and its high solubility and bioavailability allow it to easily disperse in the environment. In contrast, Cr(III) is less toxic and less soluble than Cr(VI), and it has the ability to precipitate (Guo et al. 2021; Saha et al. 2011). Reducing Cr(VI) to Cr(III) is a key strategy for the remediation of contaminated sites (Michailides et al. 2015).

Various biotic and abiotic remediation strategies have been proposed for Cr(VI) contaminated environments. Abiotic strategies include physical methods such as containment, which are inefficient in the long term, and chemical treatments that often generate toxic secondary waste, exacerbating contamination issues (Dhal et al. 2013; Mukherjee et al. 2013; Němeček et al. 2015). In contrast, bioremediation is a cost-effective and eco-friendly approach (Lara et al. 2017; Michailides et al. 2015; Song et al. 2021).

Biostimulation of indigenous bacterial consortia has emerged as a promising approach for in situ bioremediation (Joutey et al. 2011; Singh et al. 2022; Lara et al. 2017). However, the successful implementation of biostimulation largely depends on understanding the unique physicochemical attributes of each site, as the efficiency of the reduction process is strongly influenced by the indigenous microbiota (Francisco et al. 2002; Wu et al. 2022). Therefore, a thorough understanding of site-specific conditions is essential for optimizing biostimulation strategies and achieving successful bioremediation outcomes.

Metagenomic and metatranscriptomic approaches serve as powerful tools for investigating microbial diversity, enabling a deeper understanding of community structure and the molecular functions of non-cultivable microorganisms (Chai et al. 2019; Yu et al. 2021). In this sense, it is crucial to analyze the metabolic capacities of native microbiota at the beginning and during the biostimulation process. This analysis allows for the evaluation of microbial activity after the addition of an electron donor and the assessment of its efficiency to enhance the Cr(VI) reduction process.

In this study, we characterized the bacterial community structure in a site exposed to hexavalent chromium contamination for over four decades. Additionally, we inferred its metabolic potential through metagenomic analyses, implemented a successful in situ biostimulation pilot assay, and analyzed the microbial community response during the Cr(VI) reduction process using metatranscriptomic studies.

## Materials and methods

### Long-term chromate contaminated study site description, sample collection, and physicochemical parameters

The study site is located in San Francisco del Rincón, Guanajuato, Mexico. Chromium contamination in this region has been reported for more than four decades (Armienta-Hernández and Rodríguez-Castillo 1995); Brito et al. 2013; Lara et al. 2017). The main sources of contamination originate from anthropogenic activities (Armienta-Hernández and Rodríguez-Castillo 1995). Chromium concentrations exceeding 500 mg kg⁻^1^ have been recorded near an environmental liability containing more than 300 tons of Cr(VI) residues (chromite ore product residue pile, COPRP), as well as at various sites in the León, Guanajuato valley (Armienta-Hernández and Rodríguez-Castillo 1995; Lara et al. 2017).

Samples were collected 3 m away, in a southwest direction, from a COPRP (21°04′ 27″ N, 101°79′ 10″ W). Two sampling points (A and B) were selected, where 2 cm of the surface soil layer were removed. Soil samples were taken at two depths: 0–20 cm (A1, B1) and 20–40 cm (A2, B2). The samples were ground, homogenized, sieved with a #20 mesh, and stored at 4 °C until processing. Soil pH was determined as follows: 2 g of soil were mixed with 20 ml of distilled water, shaken for 1 h, and allowed to stand for 1 h before measuring the pH using an Orion* 2-Star Benchtop pH Meter (Thermo Scientific). Cr(VI) concentration was determined through a colorimetric reaction with diphenylcarbazide in acid solution, measured at 540 nm, according to the methods established by the American Public Health Association, the American Water Works Association, and the Water Environment Federation; The measurements were performed in triplicate. In order to know the soil viability and the soil fertility diagnosis A mixture of 1 kg of soil, extracted from a depth of 0 to 30 cm and at a maximum distance of 40 cm from the points where the biostimulation assays, were conducted was analyzed at Fertilab® S. de R.L., where parameters such as pH, salinity, essential elements, and exchangeable cations were quantified (Figure S5; Table S1).

### Pilot in situ biostimulation assay

An in situ biostimulation assay was conducted at two points, designated A and B, at depths of 20 and 40 cm, respectively. Sterile perforated polypropylene bags were filled with 3 kg of soil and 500 ml of 2% molasses. These bags were left in place at the A and B points for 20 days. pH and Cr(VI) concentration were determined in the laboratory from samples collected and stored at −70 °C until DNA and RNA extraction.

### Total DNA extraction

DNA was extracted from 0.5 g of soil from A1-T0, A2-T0, A1-20D and A2-20D samples, using a modified method described by Valenzuela-Encinas et al. (2008). Initially, 1 ml of 0.15 M sodium pyrophosphate solution (PP solution) was added to the sample in a 15-ml conical tube, followed by vortexing for 1 min and centrifugation at 7500 rpm for 8 min. The resulting supernatant was decanted, and this process was repeated twice (1.1 Supplementary material). DNA was precipitated overnight at 4 °C, then resuspended in deionized water and stored at −20 °C until use. The concentration and quality of the extracted DNA were determined using a Nanodrop spectrophotometer and agarose gel electrophoresis.

### 16S rDNA amplicon sequencing and shotgun metagenome sequencing

Total environmental DNA from the soil samples of point A at time zero (T0) and after 20 days (20D) of in situ biostimulation, was used to construct V3–V4 amplicon libraries, which were pooled at equimolar concentrations and purified with the MoBio Ultraclean PCR Clean-Up Kit. Paired-end sequencing of these libraries was carried out on an Illumina MiSeq platform at UUSMB UNAM (Cuernavaca, Morelos, México). Additionally, environmental DNA collected from site A at depths of 20 and 40 cm at time zero (A1-T0 and A2-T0), pooled together, was used for whole metagenome shotgun sequencing. Sequencing of paired-end reads was conducted on an Illumina NextSeq500 platform, utilizing 75 or 150 cycles per side, also at UUSMB UNAM (Cuernavaca, Morelos, México).

### Total RNA extraction and sequencing

The total RNA extraction protocol was modified from Holmes et al. (2004). Briefly, 10 g of the A1-20D sample was used. Solutions and the duration of subsequent steps were adjusted according to the sample amount (1.2 Supplementary material). RNA was precipitated overnight at −70 °C, and the resulting pellet was resuspended in 200 µl of sterile DEPC-treated water (Ambion). The resuspended pellets were purified using the RNA Clean & Concentrator kit (Zymo Research). RNA was treated with DNA-free DNase (Ambion) according to the manufacturer’s instructions. The concentration and quality of the extracted RNA were determined using a Nanodrop spectrophotometer and agarose gel electrophoresis.

Total RNA from the A1-20D sample was used for paired-end sequencing on an Illumina NextSeq500 platform with a configuration of 75 or 150 cycles per side by UUSMB UNAM (Cuernavaca, Morelos, Mexico).

### 16S rRNA bioinformatics analysis

16S rRNA data analysis was performed using QIIME2 version 2021.4. (Bolyen et al. 2019) After importing the data into QIIME2, sequences were filtered (forward and reverse reads were truncated at position 280 and 240 respectively), merged and chimeric sequences were removed using the DADA2 plugin. Taxonomic assignments of the Amplicon Sequence Variants (ASVs) were conducted through the “feature classifier” plugin with the “classify-consensus-blast” command trained on 16S rRNA gene OTUs clustered at 99% similarities within the Silva 132 database (Yilmaz et al. 2014). All redundant blast hits (identical taxonomic assignments) were collapsed using the “taxa collapse” plugin at each taxonomic level (Phylum, Class, Order, Family and Genus). Sequences that could not be allocated to the corresponding taxonomic level (but were allocated to any higher level) were accumulated into a new category named “Unassigned.”

### Metagenome and metatranscriptome analysis

The shotgun sequences obtained from the metagenome and metatranscriptome were processed using the SqueezeMeta v1.00 pipeline (Tamames and Puente-Sánchez 2019) in sequential and co-assembly mode respectively. In summary, assembly for each sample was performed using Spades (Bankevich et al. 2012); removal of short contigs (< 200 bp) and contig statistics were done using prinseq (Schmieder and Edwards 2011); gene prediction from the contigs was performed using Prodigal (Hyatt et al. 2010); 16S RNAs were predicted using Barrnap and subsequently classified using the RDP classifier (Wang et al. 2007); comparison of gene sequences against GenBank (Benson et al. 2018) for taxonomic assignment,eggNOG (Huerta-Cepas et al. 2016) for COG/NOG annotation, and KEGG (Kanehisa and Goto 2000) for KEGG ID annotation, were conducted with Diamond (Buchfink et al. 2015); gene sequences were also classified against the PFAM (Finn et al. 2014) database using HHMER3 (Eddy 2009); coverage and abundance estimation of the genes and contigs were performed with Bowtie2 (Langmead and Salzberg 2012). ORFtable generated by SqueezeMeta pipeline, was used as the primary dataset for constructing KO and genus networks, creating KEGG module heatmaps, and developing the metabolic potential cladogram.

### Networks analysis

To identify bacteria harboring genes that confer Cr(VI) resistance and reduction, as well as bacterial groups expressing key reductive genes during biostimulation, we constructed networks correlating KO numbers with the bacterial genera associated with the identified genes or transcripts.

We built two distinct networks. The first was based on KO (KEGG Orthology) (Kanehisa et al. 2025) numbers corresponding to the most prevalent reductases detected in the metatranscriptome. In parallel, we built a second network using KO numbers associated with genes conferring resistance to heavy metals. In both cases, KO annotations were linked to taxonomic information from the ORFtable, primarily at the genus level or the most specific taxonomic level available when genus-level data was absent.

After annotation, we calculated cumulative TPM (Transcripts Per Million) values for each KO, integrating data from both metagenomes and metatranscriptomes. In these networks, nodes represented either a KO or a genus, with edge width proportional to TPM values. Additionally, node color indicated data origin: blue for metagenomic and red for metatranscriptomic data. Network construction was performed using Cytoscape (version 3.6.1).

### Metabolic potential cladogram

To construct the metabolic potential cladogram, hierarchical functional annotations from Brite were retrieved from the KEGG database. These annotations were then used to generate the cladogram using Graphlan software (Asnicar et al. 2015).

## Results and discussion

### Study site description and physicochemical characterization

In this study, we conducted a pilot in situ biostimulation assay at two sampling points, A and B (Figure S4), at two different depths: 20 cm and 40 cm. Sample A at a depth of 20 cm (A1) and sample B at a depth of 20 cm (B1) showed Cr(VI) concentrations of 12,445.03 mg/kg and 7,720.31 mg/kg, respectively (Table 1). Additionally, samples A at a depth of 40 cm (A2) and B at a depth of 40 cm (B2) exhibited Cr(VI) concentrations of 6,421.01 mg/kg and 5,594.19 mg/kg, respectively (Table 1). The pH conditions at the study site ranged from 8.6 to 9.1, and during biostimulation, it became more alkaline, increasing from 9.1 to 10.2. According to the results of the soil fertility analysis, the soil is classified as sandy clay loam and has a conductivity of 12,347.5 dS/m ± 281.94 (Table 1S).

**Table 1:** Soil sample characteristics

Sample	Deep (cm)	without molasses (T0)		20 days biostimulated (T20d)	
		[Cr(VI)] mg/kg	pH	[Cr(VI)] mg/kg	pH
A1	0–20	12,445.03	8.5	3028.0848	9.1
A2	20–40	6421.012	9.1	854.7136	10.2
B1	0–20	7720.31	8.9	299.559	9.2
B2	20–40	5594.186	8.7	75.1348	9.5

The presence of such high levels of Cr(VI) not only at the study site but also in surrounding areas raises significant concerns for both the environment and public health (Henkler et al. 2010; Zeng et al. 2016; Zhitkovich 2011). The tanning industry is one of the main economic activities in the region. As a result, the processes involved in the production of chromium compounds used in tanning, along with the improper management of waste generated from these industrial activities (such as the accumulation of COPRP), have contributed to severe contamination of the nearby soil and groundwater.

### Pilot in situ biostimulation assay with molasses

The pilot in situ biostimulation assay was performed as described in the Materials and Methods section. The selection of the electron donor was previously determined through microcosm biostimulation assays using 2% molasses, 20 mM lactate, and 20 mM acetate (Figure S1). The effect of molasses on hexavalent chromium reduction has been studied in other batch experiments under different oxygen conditions (aerobic, anaerobic, and microaerophilic), with a reduction time of 5 days in all cases. The effect of sterile molasses was also compared to pasteurized molasses. The reduction of Cr(VI) in tests with pasteurized molasses was achieved in an average of 3 days. Molasses is a complex mixture of carbohydrates, primarily sucrose, glucose, and fructose (Day-Lewis and Schaffler 1992). Results suggested that pasteurized molasses, at a concentration of 2%, was the best electron donor for the indigenous microorganisms of this soil to carry out Cr(VI) reduction (Figure S1).

**Fig. 1:**
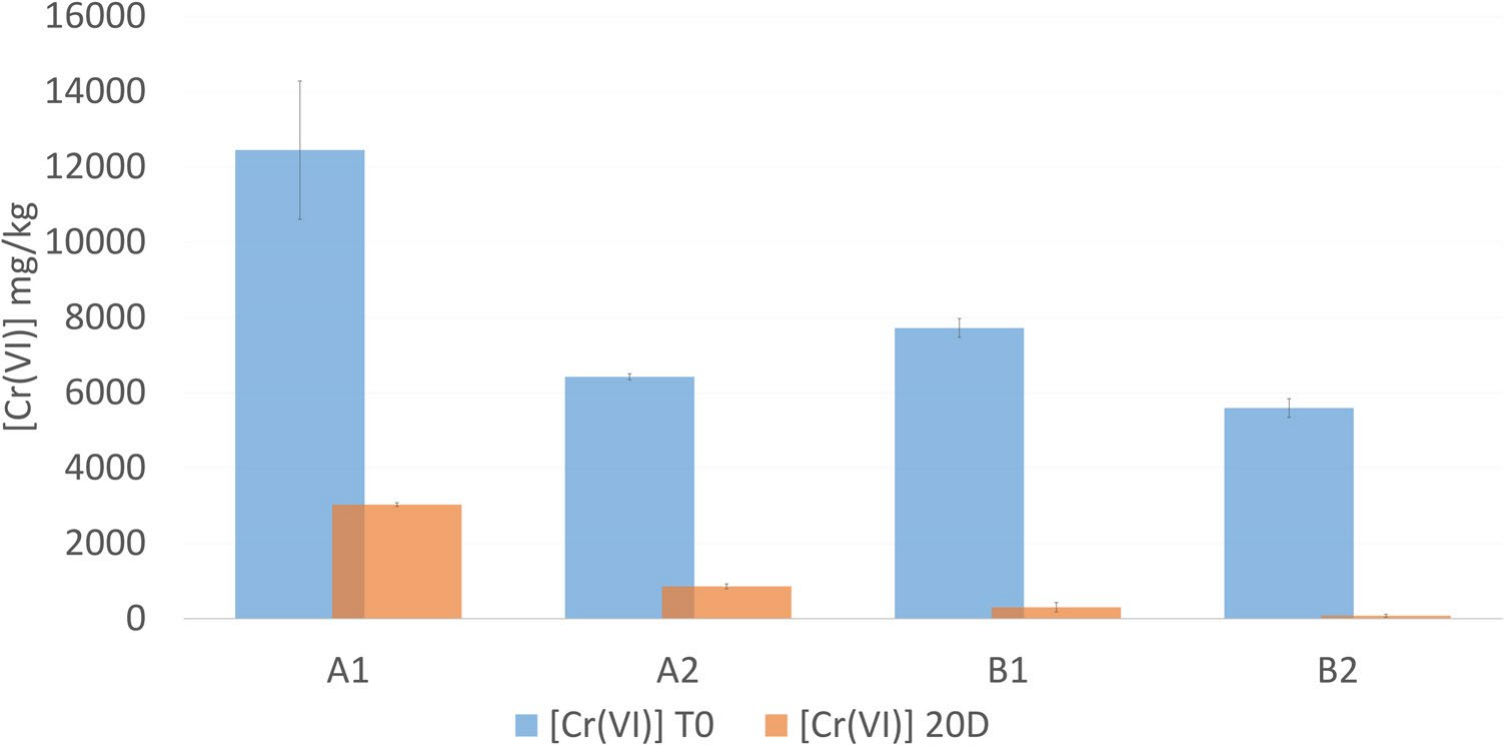
Cr(VI) reduction during in situ biostimulation pilot assay. Cr(VI) after 20 days of biostimulation. Assays with a higher initial Cr (VI) content require more days to Cr(VI) complete reduction

Samples from the pilot in situ biostimulation assay were taken at the initial time and 20 days after biostimulation. The Cr(VI) concentration at the initial time was 12,445 mg/kg and 6,421 mg/kg of soil at points A0-20 cm and A20-40 cm, respectively. After biostimulation, these concentrations decreased to 3,028.08 mg/kg and 854.71 mg/kg, respectively (Fig. 1). Samples from points B0-20 cm and B20-40 cm also showed Cr(VI) reduction, from 7,720.31 mg/kg and 5,594.19 mg/kg to 299.55 mg/kg and 75.13 mg/kg, respectively. These results indicate that in situ biostimulation with molasses is effective in reducing Cr(VI) (Fig. 1).

It has been observed that some microbial groups utilize molasses as electron donors and carbon sources, while others use it as a substrate, providing optimal conditions for Cr(VI) reduction. Previous studies have shown that a mixed culture of microorganisms indigenous to industrial sludge can use molasses as a carbon source to enhance Cr(VI) reduction (Michailides et al. 2015). Furthermore, native microbiota from other polluted sediments have adapted to resist high concentrations of Cr(VI), exceeding 12,000 ppm, and this site also possesses high salt content and a pH of 8.5 (Lara et al. 2017). In our study, the initial pH in all samples was 8.5–9, and it increased to 9–10.5 during biostimulation (Table 1). In addition to its effectiveness, molasses is a low-cost electron donor that does not produce other toxic compounds as part of its metabolism and degradation (Day-Lewis and Schaffler 1992).

### Analysis of microbial diversity before and after in situ biostimulation treatment

Bacterial diversity from soil samples was analyzed at the initial time point (T0) and after biostimulation (20 days) at site A: A1 (0–20 cm depth) and A2 (20–40 cm depth) using high-throughput sequencing of 16S rRNA gene amplicons. A total of 1,232,123 high-quality reads with an average read length of 300 bp were obtained. At the initial time point (T0), the diversity at the phylum level was as follows: *Proteobacteria* (60–38%), *Actinobacteria* (23–34%), *Firmicutes* (8–11%), *Chloroflexi* (0.24–7%), *Patescibacteria* (6–2.9%), and *Gemmatimonadetes* (2.7–2.7%) in samples A1 and A2, respectively.

**Fig. 2:**
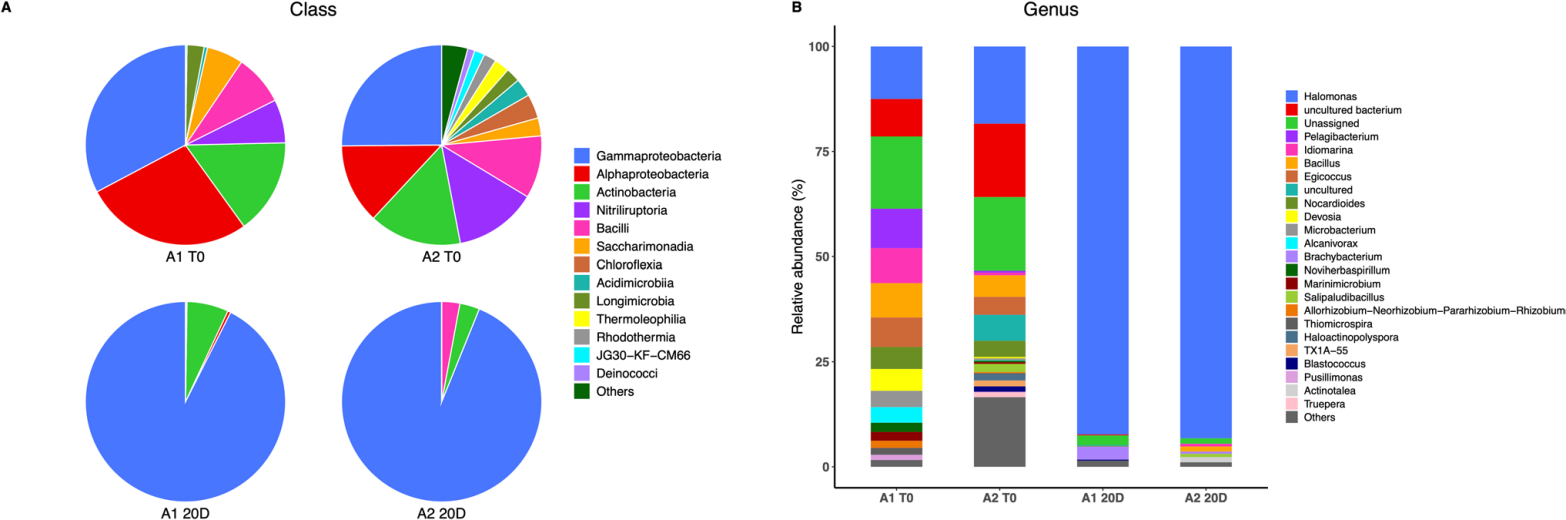
**A**) Class-level microbial diversity analysis. A1 and A2 sample “A” at 0–20 cm and 20–40 cm respectively. T0 (initial time) and 20 days biostimulation assay. **B**) Genus-level microbial diversity

The bacterial community was dominated by *Gammaproteobacteria* (32.74–25.13%), followed by *Alphaproteobacteria* (27.21–12.92%) and *Actinobacteria* (15.40–14.95%). Other present classes included *Nitriliruptoria* (7.02–13.37%), *Bacilli* (8.18–10.09%), *Saccharimonadia* (5.90–2.91%), *Chloroflexia* (0.00–3.92%), *Acidimicrobia* (0.54–2.90%), and *Longimicrobia* (2.77–2.31%) in samples A1 and A2, respectively, at T0 (Fig. 2A).

After 20 days of biostimulation with molasses, the abundance of bacterial classes changed, with *Gammaproteobacteria* (92.55–93.86%) becoming the dominant class, followed by *Alphaproteobacteria* (0.49–0.00%) and *Actinobacteria* (6.72–3.21%) in samples A1 and A2, respectively (Fig. 2A).

Comparisons with microbial community analyses from others tannery-impacted soils revealed that *Acidobacteria, Deltaproteobacteria, Alphaproteobacteria,* and *Gammaproteobacteria* are commonly prevalent and have been implicated in Cr(VI) bioreduction, particularly through FeS particle formation (Prakash et al. 2021). Similarly, studies on soils near chromate slag deposits in Hunan Province, China, identified *Betaproteobacteria, Gammaproteobacteria,* and *Firmicutes* as dominant classes, along with *Verrucomicrobia, Deltaproteobacteria, Alphaproteobacteria, Cyanobacteria, Actinobacteria, Planctomycetes, Bacteroidetes*, and *Gemmatimonadetes* (He et al. 2016).

As expected, *Proteobacteria* was the most abundant phylum. However, the dominant bacterial groups at the class level varied due to the site-specific physicochemical conditions previously mentioned. In other cases, *Gammaproteobacteria* populations decrease under extreme conditions, such as chronic chromium contamination in alkaline soils, where *Firmicutes* tend to dominate (Desai et al. 2009). However, the use of some *Gammaproteobacteria* as bioindicators of environmental alterations has been reported (Desai et al. 2009; Zhang et al. 2021).

At the genus level, at the initial time point (T0), *Halomonas* was the most abundant genus in samples A1 and A2, with a representation of 12.51% and 18.40%, respectively (Fig. 2B). Other identified genera included *Pelagibacterium* (9.37–0.48%), *Idiomarina* (8.26–0.61%), *Bacillus* (8–5%), *Egicoccus* (7.02–4.26%), and *Nocardioides* (5.24–3.8%; Fig. 2B). These bacterial groups have been reported in slightly saline environments and soils with a wide pH range (Shapovalova et al. 2009; Sorokin et al. 2009). Genera such as Halomonas and Bacillus have been isolated from contaminated environments, where some members exhibit the ability to degrade xenobiotics, including hydrocarbons and heavy metals. Additionally, certain *Halomonas* species produce osmolytes and polyhydroxybutyrate (PHB) under stress conditions (Gutiérrez et al. 2012; Shapovalova et al. 2009).

*Pelagibacterium*, a genus of *Alphaproteobacteria*, is widely distributed in marine ecosystems and oligotrophic environments (Xu et al. 2011). This genus has been detected on the surface of plastics in the landfill plastisphere, suggesting a potential role in these environments (Lin et al. 2023a, b). Its presence in soils indicates that it may contribute to organic matter decomposition and microbial resilience under adverse conditions (Yin et al. 2021; Soria et al. 2024). The genus Idiomarina includes species capable of metal accumulation and nanoparticle synthesis (Seshadri et al. 2012; Morcillo et al. 2014). Bacteria from the genus Egicoccus are known for their ability to tolerate salinity and osmotic stress, allowing them to survive in ecosystems where other bacterial species cannot thrive (Chen et al. 2021).

The *Nocardioides* genus could play a key role at the site, as some of its species have been reported to produce arsenate reductase, facilitating the conversion of the less mobile As(V) into the more soluble As(III) (Bagade et al. 2016). The identified bacterial community exhibited promising potential for Cr(VI) reduction due to its bioremediation-associated metabolic capabilities. Differences in bacterial abundance were observed between depths; however, the microbial composition suggests an adaptive response to the site's environmental conditions, potentially enabling the expression of metabolic pathways involved in contaminant transformation.

The Shannon diversity index showed a decrease in bacterial diversity during biostimulation, from 2.9–4.16 to 0.47–0.46 in samples A1 and A2, respectively, as a specific bacterial consortium was enriched. The *Halomonas* genus increased its relative abundance from 12.51 to 92.30% in A1 and from 18.4 to 93.22% in A2.

*Halomonas* isolates have been proposed for bioremediation. For instance, *Halomonas sp. M-Cr* has been suggested for Cr(VI) removal in saline and alkaline environments (Mabrouk et al. 2014). Similarly, bacterial community shifts during soil remediation from abandoned chromium salt waste in Hebei Province, China, showed that bacterial species diversity decreased, with *Bacillus spp*. and *Halomonas* spp. becoming dominant (Fu et al. 2021).

Biostimulation of the bacterial community is an effective strategy for Cr(VI) reduction. This approach has been tested in various sites and with different contaminants, often combined with complementary techniques to enhance efficiency, such as sulfate-reducing bacteria biostimulation (Yang et al. 2021), hydrocarbon contamination bioremediation (Wu et al. 2019), and pentachlorophenol bioremediation (Ammeri et al. 2022). Biostimulation is a sustainable and efficient strategy for heavy metal speciation into less toxic forms.

The high tolerance and rapid Cr(VI) reduction observed in this indigenous microbial community may be due to metabolic interactions within the consortium. Microbial consortia enrichment has been shown to enhance resistance and Cr(VI) reduction efficiency over short periods (Lin et al. 2023a, b; Zhang et al. 2022). Although bacterial diversity decreased drastically during biostimulation, its presence in contaminated soil at the initial stage suggests that these bacteria possess the necessary metabolic pathways to adapt to this hostile environment, however, the dominance of the genus Halomonas during biostimulation is particularly relevant.

### Taxonomic analysis of metagenome (MG) and metatranscriptome (MT)

To determine the metabolic potential of indigenous bacteria, we sequenced the total DNA extracted from a contaminated sample. As a first step, we performed taxonomic assignment using SqueezeMeta to validate the results obtained from the 16S rRNA analysis. At the class level, 38.52% of these sequences correspond to *Actinobacteria*, 28.20% to *Gammaproteobacteria*, 14.77% to *Nitriliruptoria*, 5.3% to *Alphaproteobacteria,* 10.97% to unclassified *Proteobacteria*, and the remainder to other bacteria (Figure S2).

This result aligns with the microbial diversity analysis, where bacteria from these phyla are observed at the initial time point. *Proteobacteria* is a widely studied phylum, whose metabolic diversity allows its members to survive even in environments highly contaminated with Cr(VI), such as industrial tanning waste effluents, toxic sludge, and industrial leachate sediments (Francisco et al. 2002; Prakash et al. 2021; Zeng et al. 2016). In other studies conducted under similar conditions, the dominant phyla are Actinobacteria, Firmicutes, and Bacteroidetes.

At 20 days of in situ biostimulation, the highest number of assigned sequences in the metatranscriptome correspond to *Gammaproteobacteria* class, specifically to the genus *Halomonas* (Figure S2).

The taxonomic assignment of other important sequences found in the metatranscriptome, which indicate the expression of genes related to Cr(VI) reduction and resistance, shows that, to a lesser extent, in addition to *Halomonas*, a select group of bacteria is carrying out key processes for molasses assimilation and Cr(VI) reduction (Figure S2). It was observed that during biostimulation with molasses, a specific bacterial consortium was enriched. These bacterial groups are commonly found in sites with a pH range of 6–10, and some of them have been characterized by their ability to resist high concentrations of salt (Ahemad 2014). It has been observed that some of these bacteria possess molecular mechanisms to resist and grow in stress environments, which could also be used for resistance to heavy metals. The tolerance and rapid reduction of high natural concentrations of Cr(VI) may result from metabolic relationships between consortium members, as it has been shown that microbial consortium enrichment is the best way to achieve efficiency in Cr(VI) resistance and reduction in a short period.

### Comparative analysis of functional assignment of MG and MT reveals important genes for Cr(VI) resistance and reduction

The shotgun sequencing of the metagenome and metatranscriptome generated 3,882,631 and 11,617,479 paired-end sequences, respectively, with an average length of 70 bp. Nucleic acid extraction was affected by environmental factors such as high Cr(VI) concentration, salinity, and alkaline pH, which have been reported as limiting factors for nucleic acid recovery using extraction kits (Wang et al. 2021). Furthermore, it has been observed that the yield of massive sequencing decreases significantly under these physicochemical conditions, even after multiple washing and purification steps of the samples (Wang et al. 2021). Nevertheless, the data obtained allowed for a general characterization of the metabolic potential of native microorganisms during the biostimulation process, comparing the metagenome from highly Cr(VI)-contaminated soils with the metatranscriptome from an in situ biostimulation pilot assay.

**Fig. 3:**
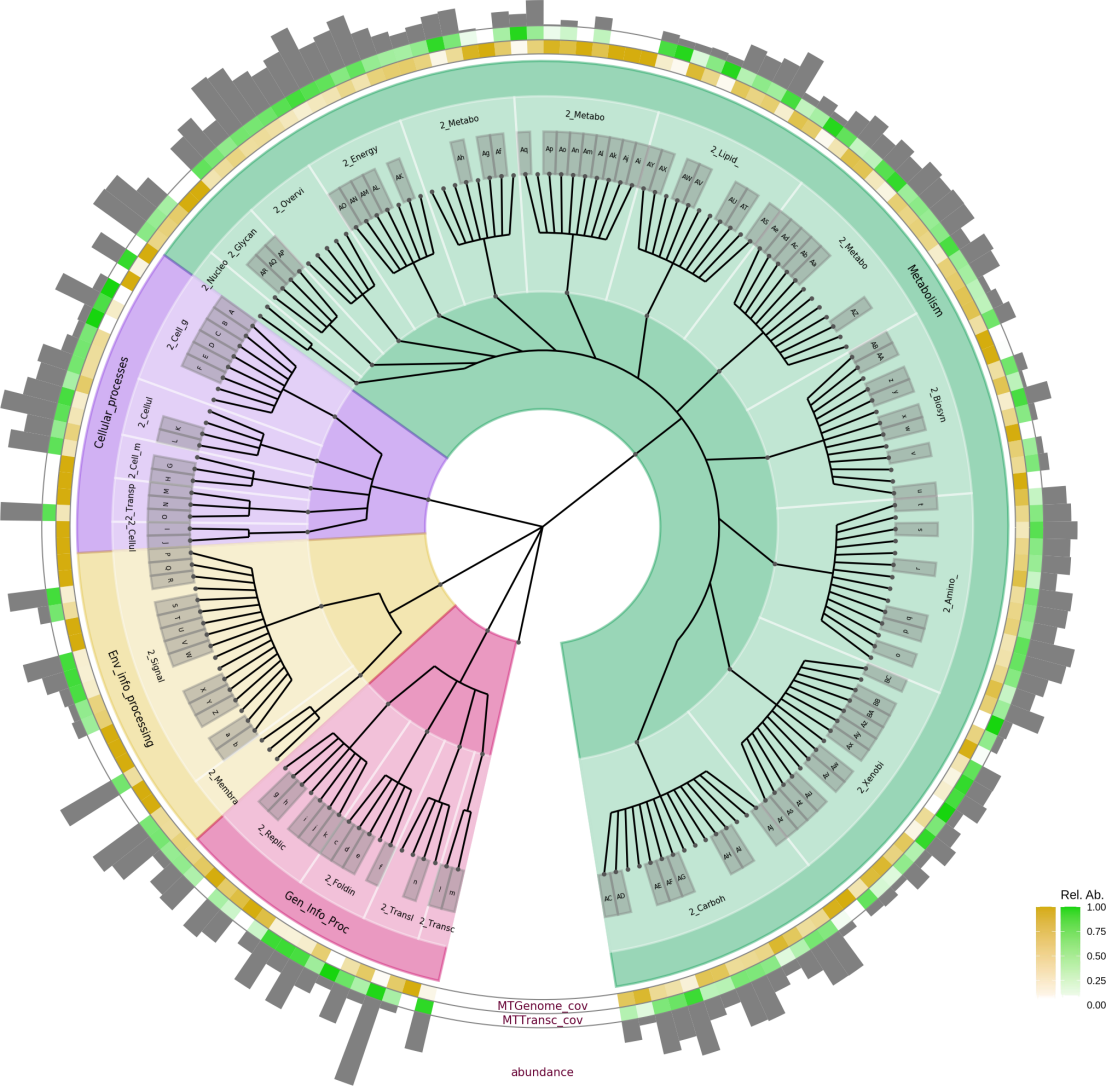
Comparative analysis of the presence and expression of genes in the metagenome (green ring; at initial time) and metatranscriptome (yellow ring; 20 days biostimulated). The bar graph is the number of transcripts in each category

The functional characterization of the metagenome revealed the presence of genes in native bacteria that have adapted for over four decades to a highly Cr(VI)-contaminated environment. On the other hand, the functional assignment of the metatranscriptome allowed the identification of active metabolic processes involved in Cr(VI) reduction during biostimulation (Fig. 3).

The functional analysis based on the KEGG database showed that most transcripts are associated with genes involved in genetic information processing, including DNA and RNA polymerases, transcriptional and translational factors, helicases, and topoisomerases. Additionally, a high expression of genes related to environmental information processing, as well as amino acid, carbohydrate, and energy metabolism, was observed (Fig. 3). These findings are consistent with previous studies suggesting that microorganisms in metal-contaminated environments frequently activate genes responsible for cellular maintenance and damage repair (Cervantes et al. 2001). This could explain the overexpression of factors associated with genetic stability observed in our study.

Furthermore, it has been established that in the presence of Cr(VI), bacteria upregulate genes involved in molecular mechanisms that mitigate oxidative stress induced by this metal (Ramírez-Díaz et al. 2008; Viti et al. 2014).

On the other hand, chromate reductases play a crucial role in the direct reduction of Cr(VI) to Cr(III), its less toxic form. These enzymes facilitate electron transfer, enabling the conversion of hexavalent chromium into a less mobile and less harmful form for microbial cells and the environment. Their activity is fundamental for biotransformation and detoxification processes in highly chromium contaminated soils (Thatoi et al. 2014).

**Fig. 4:**
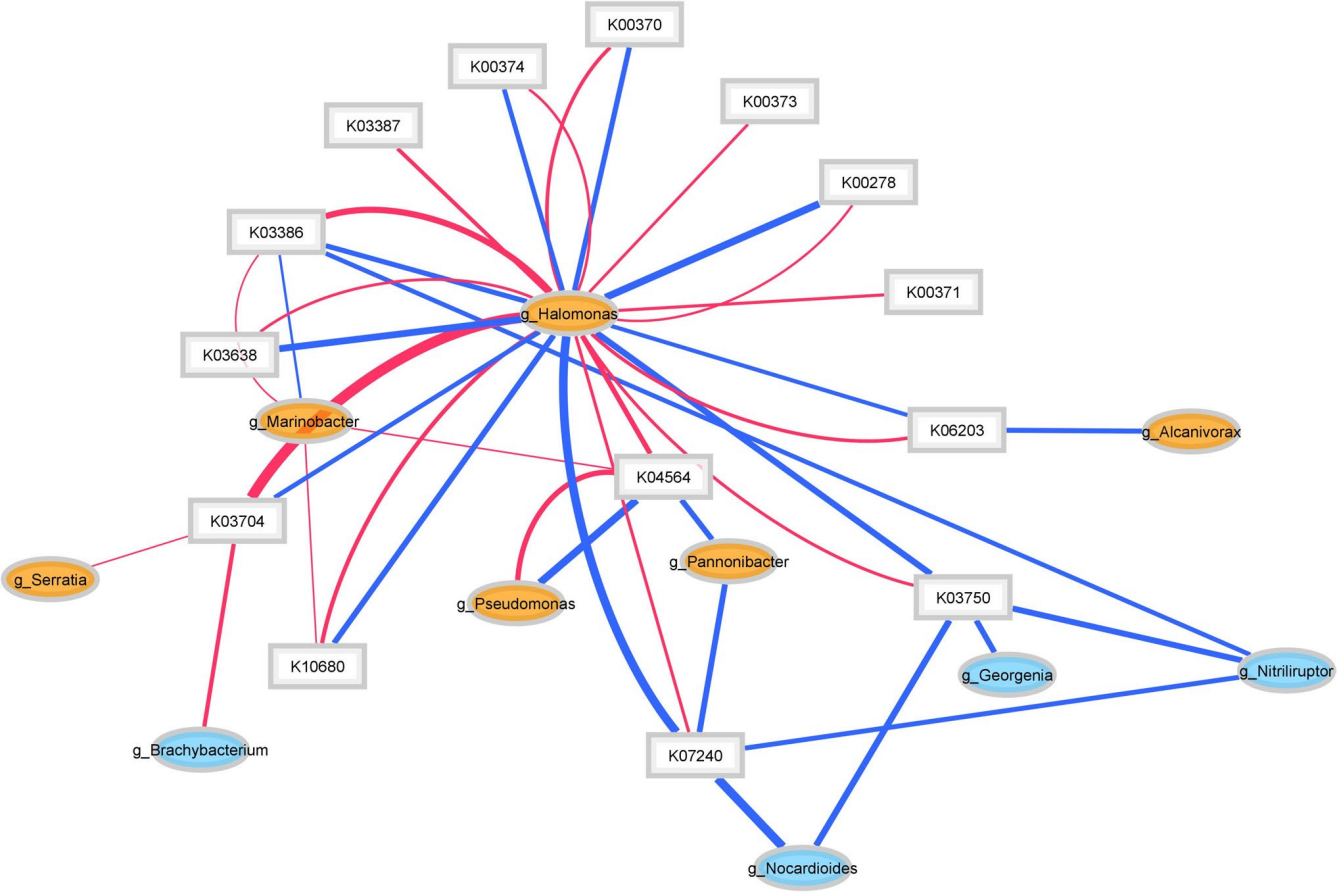
Cr(VI) resistance genes of most abundant bacteria. The blue lines are the abundance of each KO in metagenome data and the red lines are abundance in metatranscriptome. The thickness of the lines is proportional to the amount of gene expression

To further investigate the microbial mechanisms involved in Cr(VI) resistance and reduction, we selected genes related to chromium resistance and reduction for analysis in the metagenome. Subsequently, we verified whether these genes were expressed in the metatranscriptome data. Additionally, we associated these genes with bacterial genera based on the sequences they aligned with in the KEGG database (Fig. 4; Table 2).

**Table 2:** (Fig. 4) Chromium resistance genes

K number	Functions
K07240	chromate transporter
K13560	L-glutamyl-[BtrI acyl-carrier protein] decarboxylase
K01630	2-dehydro-3-deoxyglucarate aldolase
K00370	nitrate reductase/nitrite oxidoreductase, alpha subunit
K00371	nitrate reductase/nitrite oxidoreductase, beta subunit
K03750	molybdopterin molybdotransferase
K00373	nitrate reductase molybdenum cofactor assembly chaperone
K00374	nitrate reductase gamma subunit
K03638	molybdopterin adenylyltransferase,
K06203	CysZ protein
K03387	NADH-dependent peroxiredoxin subunit
K03386	peroxiredoxin
K00278	L-aspartate oxidase
K10680	N-ethylmaleimide reductase
K03704	cold shock protein

The presence of several key proteins in the metagenome highlights the microorganisms ability to resist and reduce chromium. The identification of chromate transporters (K07240) suggests that the microorganisms are equipped with mechanisms to capture chromate (Cr(VI)) from the environment, a crucial initial step in its reduction to less toxic forms (Ahemad 2014; Chromiková et al. 2022).

We found both in the metagenome and metatranscriptome the alpha (K00370), beta (K00371), and gamma (K00374) subunits of nitrate reductase, an enzyme involved in the conversion of nitrates to nitrites. Since this process may share mechanisms with Cr(VI) reduction, their presence and expression in a highly saline and alkaline environment suggest a potential role in chromium detoxification (Hu et al. 2020). The molybdenum cofactor assembly chaperone (K00373), molybdopterin molybdotransferase (K03750) and molybdopterin adenylyltransferase (K03638) are essential for the activity of these subunits, facilitating the incorporation of molybdenum into the active site of nitrate reductase (Blasco et al. 1998). The CysZ protein (K06203) is primarily involved in sulfate uptake, but some studies suggest that, due to the structural and chemical similarity between sulfate and chromium anions, this type of transporter protein could also be involved in Cr(VI) transport (Zhang et al. 2014; Su et al. 2023).

Furthermore, antioxidant proteins, such as NADH-dependent peroxiredoxin (K03387) and peroxiredoxin (K03386), help mitigate oxidative damage caused by reactive oxygen species (ROS) generated in the presence of Cr(VI) (Wang et al. 2011; Rhee et al. 2012). These proteins protect bacterial cells from the toxic effects of chromium. Likewise, the Fe–Mn family superoxide dismutase (K04564) plays a crucial role in antioxidant defense, helping maintain cellular homeostasis under chromium-induced stress (Długosz et al. 2012).

Additional proteins such as L-aspartate oxidase (K00278) and N-ethylmaleimide reductase (K10680) may be involved in the general metabolism of the cell, indirectly contributing to resistance to heavy metals, while heat shock proteins (K03704) facilitate adaptation to extreme environmental conditions associated with the presence of contaminants such as chromium (Mahmood et al. 2014).

The abundance of transcripts per million reveals a high expression of various bacteria from the *Actinobacteria* and *Gammaproteobacteria* classes in the metagenome, with *Halomonas* being predominantly expressed in the metatranscriptome (Fig. 4).

**Fig. 5:**
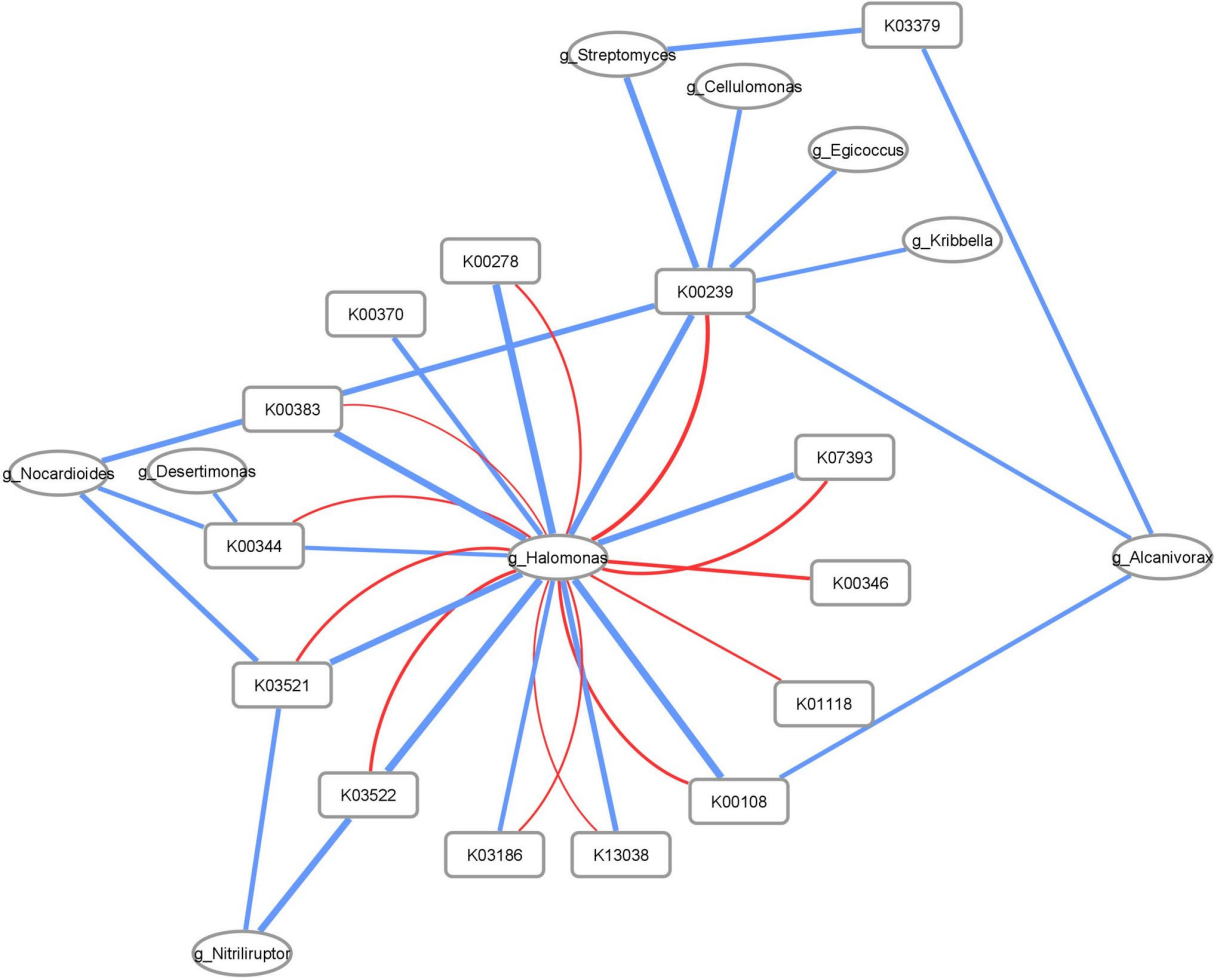
Reductases of most abundant bacteria. The blue lines are the abundance of each KO in metagenome data and the red lines are the abundance in the metatranscriptome. The thickness of the lines is proportional to the amount of gene expression

Redox potential significantly influences the reduction of Cr(VI) to Cr(III). While microorganisms play a vital role in this process, the redox environment can be affected by various factors, such as the availability of electron acceptors and donors (oxygen, iron, manganese) soil properties and even the organic matter content, which also significantly influences the efficiency and persistence of Cr(VI) reduction (Rahman and Thomas 2021). Redox reactions are crucial for chromium reduction and resistance. In metagenomic and metatranscriptomic analyses of chromium-contaminated environments, which are also highly saline and alkaline, we identified key enzymes involved in electron transfer, oxidative stress response, and metabolic adaptation. These enzymes were associated with specific bacterial taxa, shedding light on the microbial mechanisms driving chromium resistance and reduction (Table 3, Fig. 5).

**Table 3:** (Fig. 5) Reductases

K number	Functions
K00108	choline dehydrogenase
K00239	succinate dehydrogenase/fumarate reductase
K00344	NADPH:quinone reductase
K00346	Na + -transporting NADH:ubiquinone oxidoreductase
K00383	glutathione reductase (NADPH)
K01118	FMN-dependent NADH-azoreductase
K03186	flavin prenyltransferase
K03333	cholesterol oxidase
K03379	cyclohexanone monooxygenase
K03521	electron transfer flavoprotein beta subunit,
K03522	electron transfer flavoprotein alpha subunit,
K07393	glutathionyl-hydroquinone reductase
K13038	phosphopantothenoylcysteine decarboxylase
K16431	FAD-dependent halogenase
K00278	L-aspartate oxidase
K00370	nitrate reductase/nitrite oxidoreductase

Choline dehydrogenase (K00108) catalyzes the oxidation of choline to betaine, an osmoprotectant that helps counteract osmotic and oxidative stress, potentially protecting bacteria from the toxic effects of chromium (Qureshi et al. 2024). Succinate dehydrogenase/fumarate reductase (K00239), which participates in the tricarboxylic acid cycle and electron transport chain, plays a crucial role in maintaining cellular redox balance, particularly under stress induced by heavy metals like chromium (Cecchini et al. 2002).

In terms of oxidative stress reduction, NADPH:quinone reductase (K00344) reduces quinones to hydroquinones using NADPH, thus preventing the formation of reactive oxygen species (ROS) and contributing to chromium resistance. Glutathione reductase (K00383) regenerates reduced glutathione, a key antioxidant that protects cells from ROS generated by Cr(VI), while glutathionyl-hydroquinone reductase (K07393) aids in detoxifying toxic hydroquinones, enhancing the antioxidant response (Ramírez-Díaz et al. 2008; Sharma et al. 2022).

Regarding energy generation and cellular homeostasis, Na + -transporting NADH:ubiquinone oxidoreductase (K00346) helps maintain ionic and energy balance, crucial in chromium-contaminated environments (Minato et al. 2014). Although research on electron transfer flavoproteins in bacteria is still limited, it is suggested that they could play a crucial role in electron transfer during cellular respiration and in defense against oxidative stress. It is proposed that the alpha and beta subunits of this flavoprotein (K03521 and K03522) facilitate key processes related to metabolic adaptation and bacterial response to adverse conditions, although further research is needed to fully understand its function. Flavin prenyltransferase (K03186), involved in flavin modification, may influence the activity of redox enzymes that contribute to chromium reduction (O'Neill et al. 2020).

Several enzymes identified may be directly involved in Cr(VI) reduction. NADH and FMN-dependent azoreductase (K01118), primarily known for reducing azo compounds, may also reduce Cr(VI), contributing to detoxification (Misal and Gawai 2018). Nitrate reductase/nitrite oxidoreductase (K00370) has been implicated in the reduction of Cr(VI) to Cr(III), suggesting its role in chromium biotransformation (Hu et al. 2020).

Other enzymes appear to be linked to metabolic adaptation in contaminated environments. Cyclohexanone monooxygenase (K03379), involved in the degradation of organic compounds, may facilitate adaptation to pollution, while phosphopantothenoylcysteine decarboxylase (K13038), involved in coenzyme A biosynthesis, and L-aspartate oxidase (K00278), catalyzing L-aspartate oxidation, may support cellular metabolism under heavy metal stress (Zhang et al. 2019).

The identification of these enzymes in the metagenome suggests that certain microorganisms possess mechanisms for chromium resistance and reduction. Notably, in the metatranscriptome, all these enzymes aligned with genes from the genus *Halomonas,* indicating that this bacterium, besides being the most abundant during biostimulation, has activated these mechanisms of resistance and chromium reduction.

These results support the hypothesis that microorganisms in Cr(VI)-contaminated soils have developed molecular mechanisms to manage chromium toxicity, activating transport, reduction, and antioxidant defense systems to ensure survival. The association of these genes with specific bacterial genera offers a more comprehensive view of the metabolic processes involved in chromium resistance and reduction in contaminated environments.

The gene relationships with the highest potential for Cr(VI) resistance, identified in both the metagenome and metatranscriptome, reinforce the role of genera like *Halomonas*, which plays a key role during biostimulation (Fig. 5). Known for its survival in extreme environments and metal biotransformation capabilities, *Halomonas* is crucial in these processes. The pH increase observed in the biostimulation assay is consistent with the reported response of bacteria such as *Halomonas* to high levels of Cr(VI) (Cheng et al. 2016); Mabrouk et al. 2014; Naz et al. 2022), as a physiological adaptation to chromium detoxification and removal (Galisteo et al. 2024). Furthermore, metatranscriptome analysis reveals the expression of genes related to Na⁺K⁺/H⁺ antiporters, specifically K03313 gene (from NhaA family of Na⁺/H⁺ antiporters) and the expression of the K00346 gene (encoding a primary Na⁺ pump) all of those related to *Halomonas* genera, within the co-expression network derived from metagenomic and metatranscriptomic data (Fig. 5 and Table 2S).

These findings highlight the need to explore specific metabolic pathways linking central metabolic processes with contaminant reduction, which could enhance future bioremediation strategies.

## Conclusion

Based on in situ biostimulation assays, the use of molasses appears to be effective for remediation for long-term chromate contaminated sites. The use of molasses as a carbon source successfully transforms toxic and soluble Cr(VI) into less toxic and poorly soluble Cr(III), suggesting its potential as a long-term treatment strategy for chromate contaminated sites.

The analysis of microbial communities revealed both resistance and reducing capabilities, indicating that indigenous bacteria possess molecular mechanisms to withstand highly alkaline and saline conditions. Metagenomic and metatranscriptomic data highlighted the prevalence of *Halomonas* bacteria, particularly in the expression of genes associated with Cr(VI) reduction. These findings emphasize the importance of understanding metabolic interactions among *Halomonas* bacteria, pivotal in facilitating Cr(VI) reduction with molasses as an electron donor. Overall, the successful reduction of Cr(VI) in the presence of molasses demonstrates its potential for effective and sustainable remediation of long-term chromate-contaminated sites. This study sets the groundwork for further investigations and advancements in harnessing the capabilities of indigenous microbial communities for environmental remediation.

## Supplementary Information

Below is the link to the electronic supplementary material.

ESM 1 (3.04 MB DOCX)

## Data Availability

The datasets generated for this study can be found in the GenBank repositoryMetagenome SRR19621753 Metatranscriptome SRR19547931 16S SRR19621698, SRR19621696, SRR19621697, SRR19621695.
