# Supplementary material for: Microbial diversity and metabolic potential in long-term Cr(VI) polluted soil during in situ biostimulation: a pilot effective assay
Source: Environ Sci Pollut Res Int. 2025 Aug 11;32(33):19885–99. doi: 10.1007/s11356-025-36804-7 (PMC12425845; doi:10.1007/s11356-025-36804-7)
Supplement: Supplementary file 1 — (3.04 MB DOCX) [file 11356_2025_36804_MOESM1_ESM.docx]

**Supplementary material**

**1 Standardized protocols for the extraction of nucleic acids from contaminated soil**

**1.1 Protocol for DNA extraction**

Modiﬁed method of Valenzuela-Encinas (Valenzuela-Encinas et al., 2008). Initially three washes were carried out with 0.5 g of soil added 1 ml 0.15 M sodium pyrophosphate solution (PP solution) in a 15 ml falcon tube (vortex for 1 min and centrifuged at 7500 rpm for 8 min). Three more washes were carried out with 1 ml of 0.15 M sodium phosphate buffer (PB buffer; pH 8) to remove residues of sodium pyrophosphate. Pellet was re-suspended in 0.5 ml of lysis solution I (0.15 M NaCl, 0.1 M EDTA, pH 8.0, 10 mg/ml lysozyme), mixed and incubated at 37 °C for 1 h. A 0.5 ml of lysis solution II (0.1 M NaCl, 0.5 M Tris–HCl, pH 8.0, 12% SDS) was added. The soil suspension was freezing at -70C for 30 min and incubated at 70 °C for 30 min and then centrifuged at 7500 rpm for 10 min. The supernatant was transferred to a 1.5-ml tube and mixed with 1/5 vol. of EDTA (0.5 M pH 8) and 1/10 vol. of potassium acetate (5 M pH 5) and incubated for 10 min at -4C. Tubes were centrifuged at 13000 rpm for 10 min and supernatant was transferred to clean 1.5 ml tube. 400 µl of Chloroform:isoamyl alcohol (24:1) was added, mixed and centrifuged for 10 min at 13 000 rpm. The supernatant was transferred to a clean tube. An equal volume of 13% PEG (polyethylene glycol [8,000 MW] dissolved in 1.6 M NaCl) was added and incubated at 4 °C overnight. Samples were centrifuged at 13000 rpm at 4C for 10 min. Pellets were washed with one volume of 70% cold ethanol and air-dried. DNA pellet was re-suspended in 50 µl of deionized water and stored at -20C until required.

- 1. **Protocol for RNA extraction**

Protocol for RNA extraction modiﬁed from Holmes et al (2004) 10g of A1_20D sample was used. 10 ml of Tris-fosfate-EDTA buffer were added and tubes were mixed and centrifuged at 5500 rpm during 5 min. The flow was discarded and 2ml of TMP buffer with 10 ml of cold acetone (stored at -70°C) were added to sediment suspensions. Tubes were mixed manually 20 times and centrifuged at 8500 rpm for 5 min. The supernatant was discarted, and 2 µl of RiboLock RNase Inhibitor (Thermo Fisher Scientific) was added to the pellet and resuspended in 5 ml of sterile DEPC-treated water (Ambion). 200 µl of lysozyme (50 mg/ml), 30 µl of proteinase K (20 mg/ml), and 600 µl of 10% sodium dodecyl sulfate solution were added and incubated at 37°C for 15 min. Samples were centrifuged at 8500 rpm for 20 min, and the supernatant was transferred to new 50-ml tubes. 200 µl of TPM buffer, 3 ml of hot acidic phenol (70°C; pH 4.5; Ambion), and 2 ml of chloroform-isoamyl alcohol (24:1) were added to the supernatant. Tubes were mixed on a Labquake rotator for 15 min and centrifuged at 8500 rpm for 5 min. The aqueous phase was removed and transferred to new 50-ml tubes, and 3 ml of hot acidic phenol (70°C; pH 4.5; Ambion) and 2 ml of chloroform-isoamyl alcohol (24:1) were added. Tubes were mixed on a rotator for 5 min and centrifuged at 8500 rpm for 5 min. The aqueous phase was removed and transferred to a new tube. 500 µl of 5 M ammonium acetate, 5 µl of Glycogen RNA grade (Thermo Fisher Scientific) and 1 vol of cold isopropanol (-20°C) were added. Nucleic acids were precipitated at -70°C overnight and centrifuged at 8500 rpm for 1.5 h. The pellet was washed with cold ethanol at 70%( -20°C) , dried and resuspended in sterile DEPC-treated water (Ambion). The resuspended pellets were cleaned with the RNA Clean & Concentrator kit (Zymo Research). RNA was treated with DNA-free DNase (Ambion) according to the manufacturer’s instructions. Concentration and quality of the extracted RNA were determined using a Nanodrop spectrophotometer and electrophoresis agarose gel.

1. **Supplementary figures**


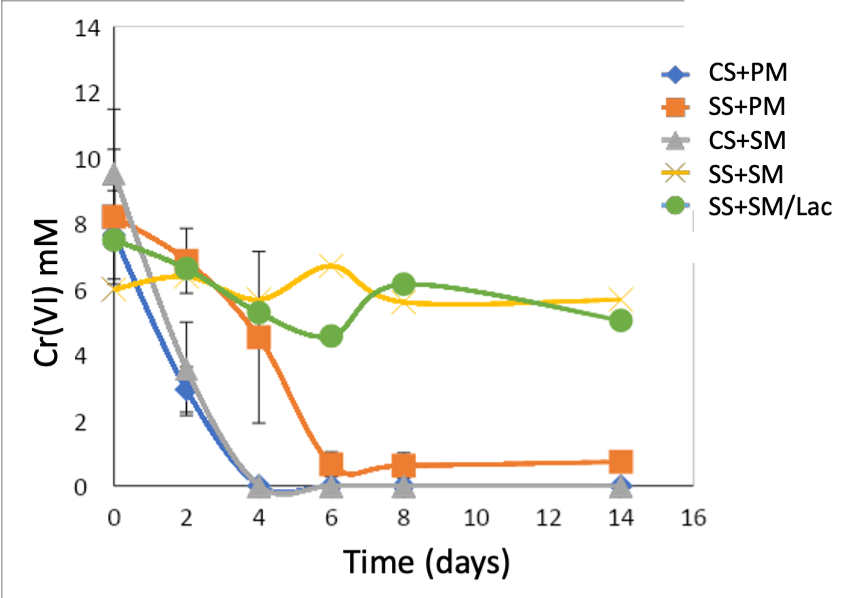


Figure S1. Cr(VI) reduction with different electron donors.- CS : Contaminated Soil, PM: pasteurized molasses, SS: sterile soil, SM : sterile molasses, SM/lac: sterile molasses with lactate.

The sterile-lactate molasses assay shows a reduction within 2 days, being the most favorable among all molasses assays, followed by pasteurized molasses and sterile molasses in non-sterilized soil. Although a reduction is also observed in pasteurized molasses with sterile soil. Soil sample from 30-40 cm depth, fourth sample.


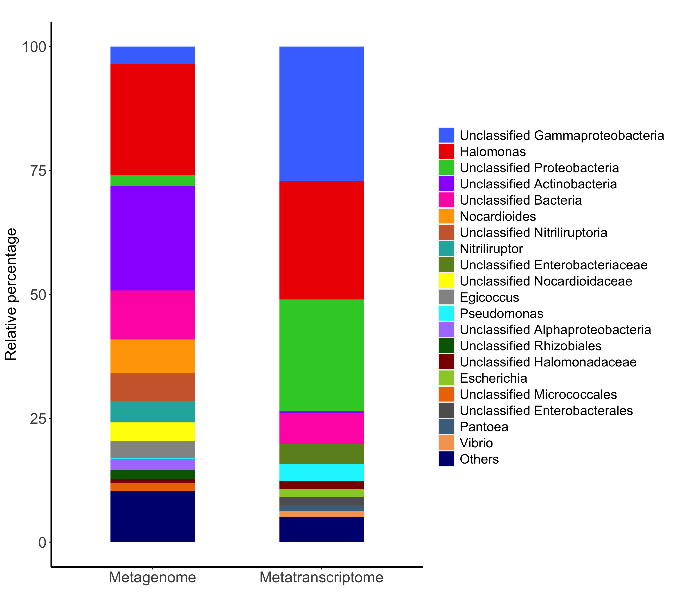

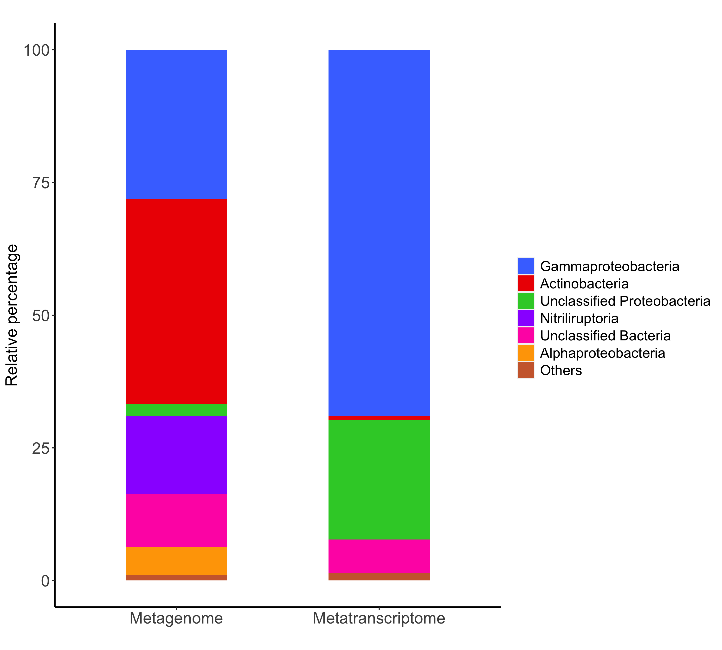


Figure S2. Taxonomic assignation of metagenome and metatranscriptome


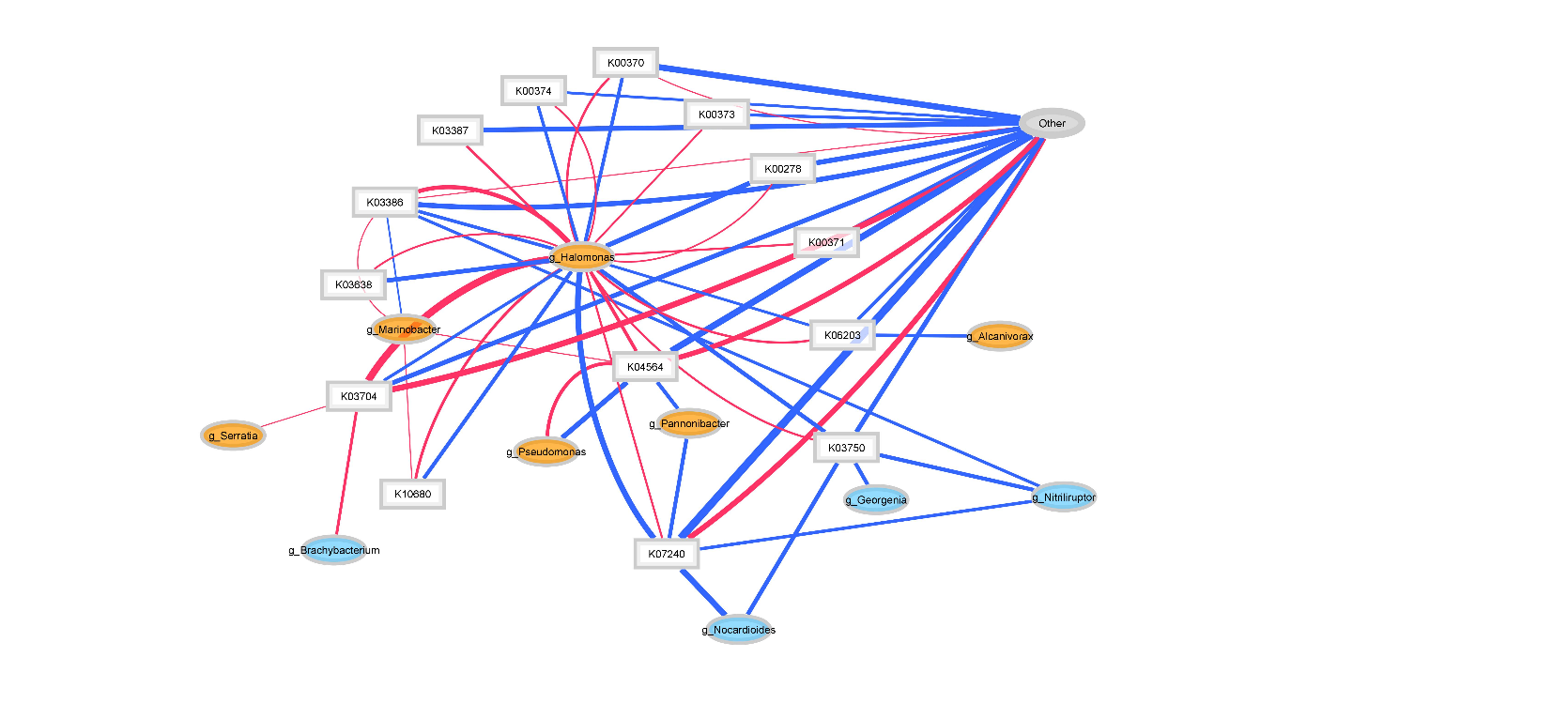


Figure S3. Reductases of different genera expressed in metatranscritome with category “Others”. This category include taxa al different assigned level

**a)**
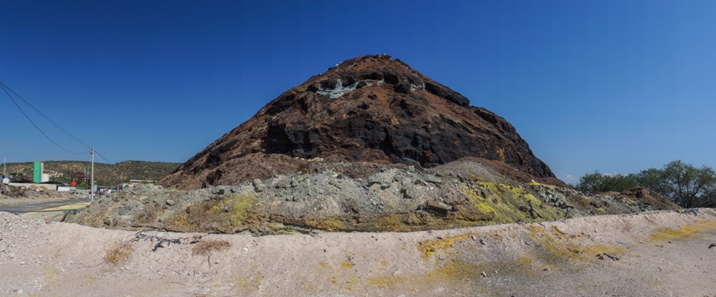


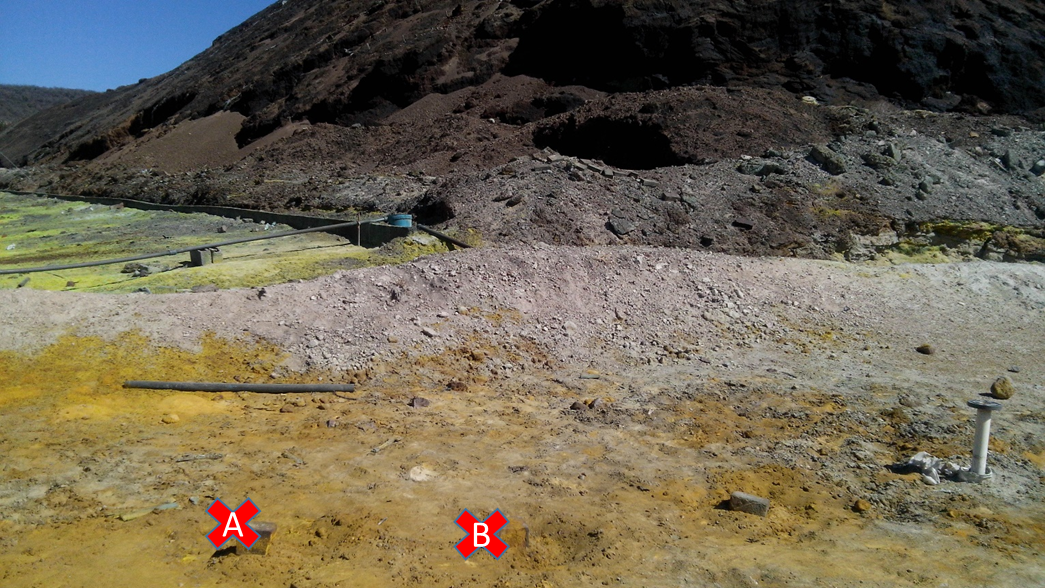


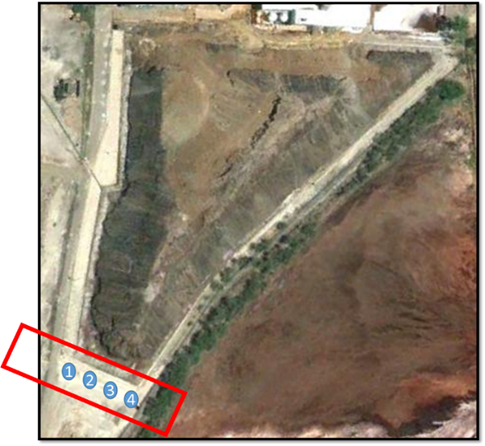


**b) c)**

Figure S4. Study site a) Frontal image of the chromium waste mountain, b) Satellite image of the chromium waste mountain with study points over time, c) Study sites for the present work


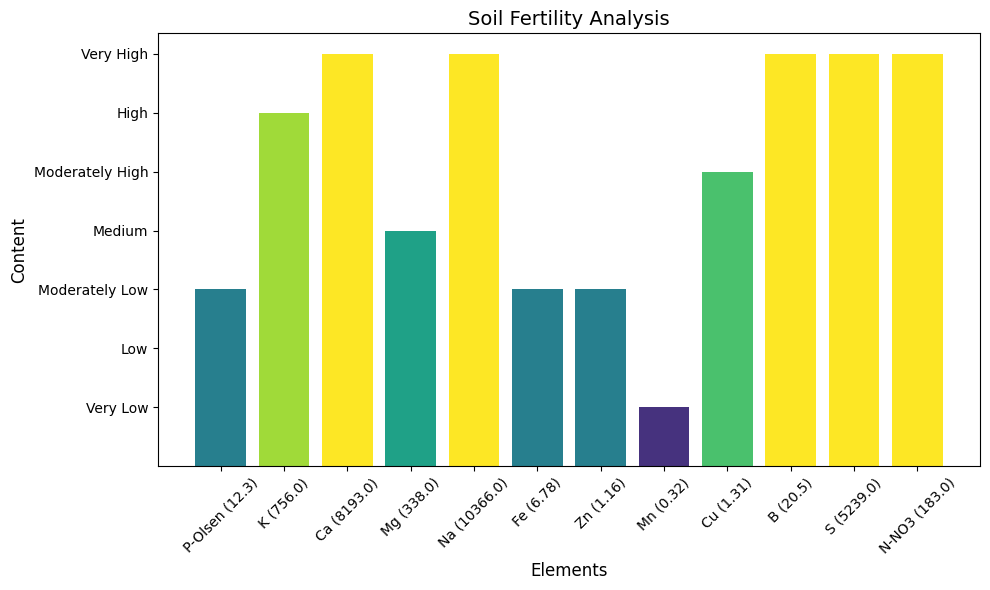


Figure S5.

1. **Supplementary tables**

| **Table 1S. Soil Fertility Analysis** | |
| --- | --- |
| **Element** | **Value (ppm)** |
| P-Olsen | 12.3 |
| K | 756 |
| Ca | 8193 |
| Mg | 338 |
| Na | 10366 |
| Fe | 6.78 |
| Zn | 1.16 |
| Mn | 0.32 |
| Cu | 1.31 |
| B | 20.5 |
| S | 5239 |
| N-NO3 | 183 |

**Table 2S. Antiporters identified in the *Halomonas* genus based on functional annotation of metatranscriptomic data**

| **Gene Name** | **COG Function** | **KEGG Function** | **Taxonomy** | **KO Identifier** |
| --- | --- | --- | --- | --- |
| cvrA, nhaP2 | NhaP-type Na+/H+ and K+/H+ antiporters with a unique C-terminal domain | cell volume regulation protein A | g_Halomonas | K03313 |
|  | Na+/H+ antiporter NhaD and related arsenite permeases |  | g_Halomonas | NA |
|  | Na+/H+ antiporter NhaD and related arsenite permeases |  | g_Halomonas | NA |
|  | Na+/H+ antiporter NhaD and related arsenite permeases |  | g_Halomonas | NA |
|  | Na+/H+ antiporter NhaD and related arsenite permeases |  | g_Halomonas | NA |
| mnhD, mrpD | Formate hydrogenlyase subunit 3/Multisubunit Na+/H+ antiporter, MnhD subunit | multicomponent Na+:H+ antiporter subunit D | g_Halomonas | K05575 |
|  | Na+/H+ antiporter NhaD and related arsenite permeases |  | g_Halomonas | NA |
| mnhD, mrpD | Formate hydrogenlyase subunit 3/Multisubunit Na+/H+ antiporter, MnhD subunit | multicomponent Na+:H+ antiporter subunit D | g_Halomonas | K05575 |
|  | Na+/H+ antiporter NhaD and related arsenite permeases |  | g_Halomonas | NA |
|  | Na+/H+ antiporter NhaD and related arsenite permeases |  | g_Halomonas | NA |
| mnhA, mrpA |  | multicomponent Na+:H+ antiporter subunit A | g_Halomonas | NA |
| mnhA, mrpA |  | multicomponent Na+:H+ antiporter subunit A | g_Halomonas | NA |
| mnhC, mrpC | Multisubunit Na+/H+ antiporter, MnhC subunit | multicomponent Na+:H+ antiporter subunit C | g_Halomonas | K05575 |
| mnhB, mrpB | Multisubunit Na+/H+ antiporter, MnhB subunit | multicomponent Na+:H+ antiporter subunit B | g_Halomonas | K05575 |
| cvrA, nhaP2 | NhaP-type Na+/H+ and K+/H+ antiporters with a unique C-terminal domain | cell volume regulation protein A | g_Halomonas | K03313 |
| cvrA, nhaP2 | NhaP-type Na+/H+ and K+/H+ antiporters with a unique C-terminal domain | cell volume regulation protein A | g_Halomonas | K03313 |
| mnhF, mrpF | Multisubunit Na+/H+ antiporter, MnhF subunit | multicomponent Na+:H+ antiporter subunit F | g_Halomonas | K05575 |
| mnhD, mrpD | Formate hydrogenlyase subunit 3/Multisubunit Na+/H+ antiporter, MnhD subunit | multicomponent Na+:H+ antiporter subunit D | g_Halomonas | K05575 |
|  | Na+/H+ antiporter NhaD and related arsenite permeases |  | g_Halomonas | NA |
| mnhA, mrpA |  | multicomponent Na+:H+ antiporter subunit A | g_Halomonas | NA |
| mnhA, mrpA |  | multicomponent Na+:H+ antiporter subunit A | g_Halomonas | NA |
| mnhA, mrpA |  | multicomponent Na+:H+ antiporter subunit A | g_Halomonas | NA |
|  | Na+/H+ antiporter NhaD and related arsenite permeases |  | g_Halomonas | NA |
